# Supplementary material for: Patterns of Intron Gain and Loss in Fungi
Source: PLoS Biol. 2004 Nov 30;2(12):e422. doi: 10.1371/journal.pbio.0020422 (PMC532390; doi:10.1371/journal.pbio.0020422)
Supplement: Table S1 — Also available at http://genes.mit.edu/NielsenEtAl/. (4.3 MB ZIP). [file pbio.0020422.st001.zip › NielsenEtAl/html/1236.html]

AN5634.1.NCU04230.1.MG04895.1.FG09896.1


```
 CLUSTAL W (1.82) Multiple Sequence Alignments - Introns Inserted


Sequence 1: NCU04230.1	548 aa
Sequence 2: FG09896.1	546 aa
Sequence 3: MG04895.1	547 aa
Sequence 4: AN5634.1	538 aa
Alignment Length: 550 aa
Number Identitical Residues: 375 aa
Alignment Score (without introns) 16635


MG04895.1 	MASKNMVNPAVEPSMEDDLFAREVAEVKQWWSDPRWRYTKRPFTAEQIVSKRGNLKIEYP
NCU04230.1	MAANNMVNPAVDPALEDELFAKEVEEVKKWWSDSRWRQTKRPFTAEQIVSKRGNLKIEYA
FG09896.1 	MASQNMTNPSINPDIEDELFQKEVEAVKTWWSDSRWRQTKRPFTAEQIVSKRGYLPIDYA
AN5634.1  	MS---------YIEEEDQRYWDEVAAVKNWWKDSRWRYTKRPFTAEQIVAKRGNLKIEYP
          	*:             **: :  **  ** **.*.*** ***********:*** * *:*.

MG04895.1 	SNAQSKKLWKILEGRFQ0KRDASYTYGCLEPTMVTQMAKYLDTVYVSGWQSSSTASSSDE
NCU04230.1	SNAQAKKLWKILEDRFA0KRDASYTYGCLEPTMVTQMAKYLDTVYVSGWQSSSTASSSDE
FG09896.1 	SNTQAKKLWKILEHRFE0NRDASYTYGCLEPTMVTQMAKYLDTVYVSGWQSSSTASASDE
AN5634.1  	SNVQAKKLWGILERNFK0NKEASFTYGCLDPTMVTQMAKYLDTVYVSGWQSSSTASSTDE
          	**.*:**** *** .*  :::**:*****:**************************::**

MG04895.1 	PGPDLADYPY0TTVPNKVSHLFMAQLFHDRKQRHERLSAPKSERSKLQNIDYLRPIIADA
NCU04230.1	PGPDLADYPY0TTVPNKVGHLFMAQLFHDRKQRQERLSVPKDQREKLANIDYLRPIVADA
FG09896.1 	PGPDLADYPY0TTVPNKVGHLFMAQLFHDRKQRQERLSVPKEQRANLLNIDYLRPIVADA
AN5634.1  	PSPDLADYPM0NTVPNKVNHLWMAQLFHDRKQREERMTTPKDQRHKVANVDYLRPIIADA
          	*.*******  .******.**:***********.**::.**.:* :: *:******:***

MG04895.1 	DTGHGGLTAVMKLTKLFIEKGAAGIHIEDQAPGTKKCGHMAGKVLVPISEHINRLVAIRA
NCU04230.1	DTGHGGLTAVMKLTKLFIEKGAAGIHIEDQAPGTKKCGHMAGKVLVPIQEHINRLVAIRA
FG09896.1 	DTGHGGLTAVMKLTKLFIEKGAAGIHIEDQAPGTKKCGHMAGKVLVPIQEHINRLVAIRA
AN5634.1  	DTGHGGLTAVMKLTKLFVERGAAGIHIEDQAPGTKKCGHMAGKVLVPISEHINRLVAIRA
          	*****************:*:****************************.***********

MG04895.1 	QADIMG1VDLLAIARTDAEAATLITTSIDPRDHAFILGCTNPSLQPLADLMNTAEQSGKT
NCU04230.1	QADIMG~SDLLCIARTDAEAATLITTTIDPRDHAFILGCTNPDLEPLADLMMKAEAEGKT
FG09896.1 	QADIMG1SDLLAIARTDAEAATLLSTNIDPRDHAFILGSTNSTLKPLNDLMIAAEATGKS
AN5634.1  	QADIMG~TDLLAIARTDSEAATLITSTIDHRDHPFIIGSTNPDIQPLNDLMVMAEQAGKN
          	******  ***.*****:*****:::.** ***.**:*.**. ::** ***  **  **.

MG04895.1 	GDQLQAIEDEWMAKANLKRFDDAVVDVINSSSSIRNPKDVAAKYLQAAKGK--SNREARA
NCU04230.1	GAQLQAIEDDWLAKADLKRFDEAVLDVIAKG-KFSNAKDLAAKYQAAVKGKQISNREARA
FG09896.1 	GAELQRIEDEWLAKANLSSFDDAVAAAIDAG-SFSDKAGIKQEYTSRAKGK--SNFEARA
AN5634.1  	GAELQAIEDEWLAKAGLKLFNDAVVDAINNS-PLPNKKAAIEKYLTQSKGK--SNLEARA
          	* :** ***:*:***.*. *::**  .*  .  : :      :*    ***  ** ****

MG04895.1 	IASSLGVPEIFFDWDSPRTREGYFRIKGGCDCAINRAIAYAPYADAIWMESKLPDYEQAK
NCU04230.1	IARQLLGQEIFFDWESPRTREGYYRLKGGCDCSINRAISYAPYCDAIWMESKLPDYAQAE
FG09896.1 	VARQLLGRDIFFDWDAPRTREGYFRLKGGCDCAVNRAIAYAPYCDAIWMESKLPDFAQAE
AN5634.1  	IAKEIAGTDIYFDWEAPRTREGYYRYQGGTQCAINRAVAYAPFADLIWMESKLPDYKQAK
          	:* .:   :*:***::*******:* :** :*::***::***:.* *********: **:

MG04895.1 	EFAEGVHAVYPEQK2LAYNLSPSFNWKTAMPRDEQETYIRRLAGLGYCWQFITLAGLHTT
NCU04230.1	EFAKGVHAVWPEQK~LAYNLSPSFNWKTAMGRDDQETYIRRLAKLGYCWQFITLAGLHTT
FG09896.1 	QFAQGVHAVWPEKK2LAYNLSPSFNWKTAMPRDEQETYIRRLAKLGYCWQFITLAGLHTT
AN5634.1  	EFADGVHAVWPEQK~LAYNLSPSFNWKKAMPRDEQETYIKRLGALGYAWQFITLAGLHTT
          	:**.*****:**:* ************.** **:*****:**. ***.************

MG04895.1 	ALISDRFARAYSEVGMRAYGELVQEPEMELGVDVVKHQKWSGATYVDELQKMVTGGVSST
NCU04230.1	ALISDQFAKAYSKIGMRAYGELVQEPEIDNGVDVVKHQKWSGATYVDELQKMVTGGVSST
FG09896.1 	ALISDQFAKAYSTVGMRAYGELVQEPEMDQKVDVVKHQKWSGATYVDELQKMVTGGISST
AN5634.1  	ALISDTFAKAYAKQGMRAYGELVQEPEMANGVDVVTHQKWSGANYVDNMLKMITGGVSST
          	***** **:**:  *************:   ****.*******.***:: **:***:***

MG04895.1 	AAMGKGVTEDQFH-
NCU04230.1	AAMGKGVTEDQFH-
FG09896.1 	AAMGAGVTEDQFK-
AN5634.1  	AAMGKGVTEDQFKS
          	**** *******::
```
